# Efficacy of lenvatinib for unresectable hepatocellular carcinoma based on background liver disease etiology: multi-center retrospective study

**DOI:** 10.1038/s41598-021-96089-x

**Published:** 2021-08-17

**Authors:** Atsushi Hiraoka, Takashi Kumada, Toshifumi Tada, Joji Tani, Kazuya Kariyama, Shinya Fukunishi, Masanori Atsukawa, Masashi Hirooka, Kunihiko Tsuji, Toru Ishikawa, Koichi Takaguchi, Ei Itobayashi, Kazuto Tajiri, Noritomo Shimada, Hiroshi Shibata, Hironori Ochi, Kazuhito Kawata, Satoshi Yasuda, Hidenori Toyoda, Tomoko Aoki, Takaaki Tanaka, Hideko Ohama, Kazuhiro Nouso, Akemi Tsutsui, Takuya Nagano, Norio Itokawa, Taeang Arai, Tomomi Okubo, Michitaka Imai, Yohei Koizumi, Shinichiro Nakamura, Koji Joko, Yoichi Hiasa, Masatoshi Kudo, The Real-life Practice Experts for HCC (RELPEC) Study Group and HCC 48 Group (hepatocellular carcinoma experts from 48 clinics in Japan)

**Affiliations:** 1 Gastroenterology Center, Ehime Prefectural Central Hospital, Kasuga-cho 83, Ehime, Japan; 2 Department of Nursing, Gifu Kyoritsu University, Ogaki, Japan; 3 Department of Internal Medicine, Himeji Red Cross Hospital, Hyogo, Japan; 4 Department of Gastroenterology and Hepatology, Kagawa University, Kagawa, Japan; 5 Department of Gastroenterology, Okayama City Hospital, Okayama, Japan; 6 Department of Gastroenterology, Osaka Medical College, Osaka, Japan; 7 Division of Gastroenterology and Hepatology, Department of Internal Medicine, Nippon Medical School, Tokyo, Japan; 8 Department of Gastroenterology and Metabology, Ehime University Graduate School of Medicine, Ehime, Japan; 9 Center of Gastroenterology, Teine Keijinkai Hospital, Sapporo, Japan; 10 Department of Gastroenterology, Saiseikai Niigata Hospital, Niigata, Japan; 11 Department of Hepatology, Kagawa Prefectural Central Hospital, Takamatsu, Japan; 12 Department of Gastroenterology, Asahi General Hospital, Asahi, Japan; 13 Department of Gastroenterology, Toyama University Hospital, Toyama, Japan; 14 Division of Gastroenterology and Hepatology, Otakanomori Hospital, Kashiwa, Japan; 15 Department of Gastroenterology, Tokushima Prefectural Central Hospital, Tokushima, Japan; 16 Hepato-Biliary Center, Matsuyama Red Cross Hospital, Matsuyama, Japan; 17 Department of Hepatology, Hamamatsu University School of Medicine, Hamamatsu, Japan; 18 Department of Gastroenterology and Hepatology, Ogaki Municipal Hospital, Gifu, Japan; 19 Department of Gastroenterology and Hepatology, Faculty of Medicine, Kindai University, Osaka, Japan

**Keywords:** Cancer, Gastroenterology, Oncology

## Abstract

It was recently reported that hepatocellular carcinoma (HCC) patients with non-alcoholic steatohepatitis (NASH) are not responsive to immune-checkpoint inhibitor (ICI) treatment. The present study aimed to evaluate the therapeutic efficacy of lenvatinib in patients with non-alcoholic fatty liver disease (NAFLD)/NASH-related unresectable-HCC (u-HCC). Five hundred thirty u-HCC patients with Child–Pugh A were enrolled, and divided into the NAFLD/NASH (n = 103) and Viral/Alcohol (n = 427) groups. Clinical features were compared in a retrospective manner. Progression-free survival (PFS) was better in the NAFLD/NASH than the Viral/Alcohol group (median 9.3 vs. 7.5 months, P = 0.012), while there was no significant difference in overall survival (OS) (20.5 vs. 16.9 months, P = 0.057). In Cox-hazard analysis of prognostic factors for PFS, elevated ALT (≥ 30 U/L) (HR 1.247, P = 0.029), modified ALBI grade 2b (HR 1.236, P = 0.047), elevated AFP (≥ 400 ng/mL) (HR 1.294, P = 0.014), and NAFLD/NASH etiology (HR 0.763, P = 0.036) were significant prognostic factors. NAFLD/NASH etiology was not a significant prognostic factor in Cox-hazard analysis for OS (HR0.758, P = 0.092), whereas AFP (≥ 400 ng/mL) (HR 1.402, P = 0.009), BCLC C stage (HR 1.297, P = 0.035), later line use (HR 0.737, P = 0.014), and modified ALBI grade 2b (HR 1.875, P < 0.001) were significant. Lenvatinib can improve the prognosis of patients affected by u-HCC irrespective of HCC etiology or its line of treatment.

## Introduction

Molecular targeted agents (MTAs) have recently been introduced for unresectable hepatocellular carcinoma (u-HCC), with sorafenib developed first in 2009 as a first-line MTA based on results presented in the SHARP^1^ and Asia–Pacific^2^ trials. Following development of that drug, lenvatinib received approval as another first-line treatment in 2018^3^. Moreover, atezolizumab plus bevacizumab treatment (Atezo + Bev), an immune-checkpoint inhibitor (ICI) and anti-vascular endothelial growth factor (anti-VEGF) combination, was recently introduced in September 2020 as a first-line treatment option for u-HCC^4^.

Despite the good therapeutic response noted for Atezo + Bev in the IMbrave 150 trial, a recent report noted that therapeutic responses to ICI treatments differed according to the etiology of the background liver disease^5^. A meta-analysis of findings in that study indicated that patients with HCC with a viral etiology showed therapeutic benefits from ICI use [HR 0.64], whereas those with a nonviral etiology did not [HR 0.92] (P = 0.03). Most importantly, results obtained in that investigation of two validation cohorts treated with ICI clearly showed that overall survival (OS) for non-alcoholic fatty liver disease or non-alcoholic steatohepatitis (NAFLD/NASH)-related HCC patients was significantly worse than that for the non-NAFLD/NASH-related HCC group (11.0 vs. 5.4 months, P = 0.023 and 17.7 vs. 8.8 months, P = 0.034, respectively). Those striking epoch-making results showed that ICI treatment response differs depending on background liver disease etiology, and especially that NAFLD/NASH-related HCC patients lack immune response as well as immune surveillance related to tumor-associated antigens.

Lenvatinib, which was approved after showing non-inferior therapeutic efficacy as compared to sorafenib, has recently come to play a large role as a first-line MTA drug in clinical practice throughout the world for u-HCC cases. However, therapeutic response in non-viral u-HCC patients given lenvatinib, especially those with NAFLD/NASH-related HCC, has not been adequately elucidated. This study aimed to evaluate differences among background hepatic disease etiology factors for therapeutic response in patients treated with lenvatinib.

## Materials and methods

### Patients

**Figure 1:**
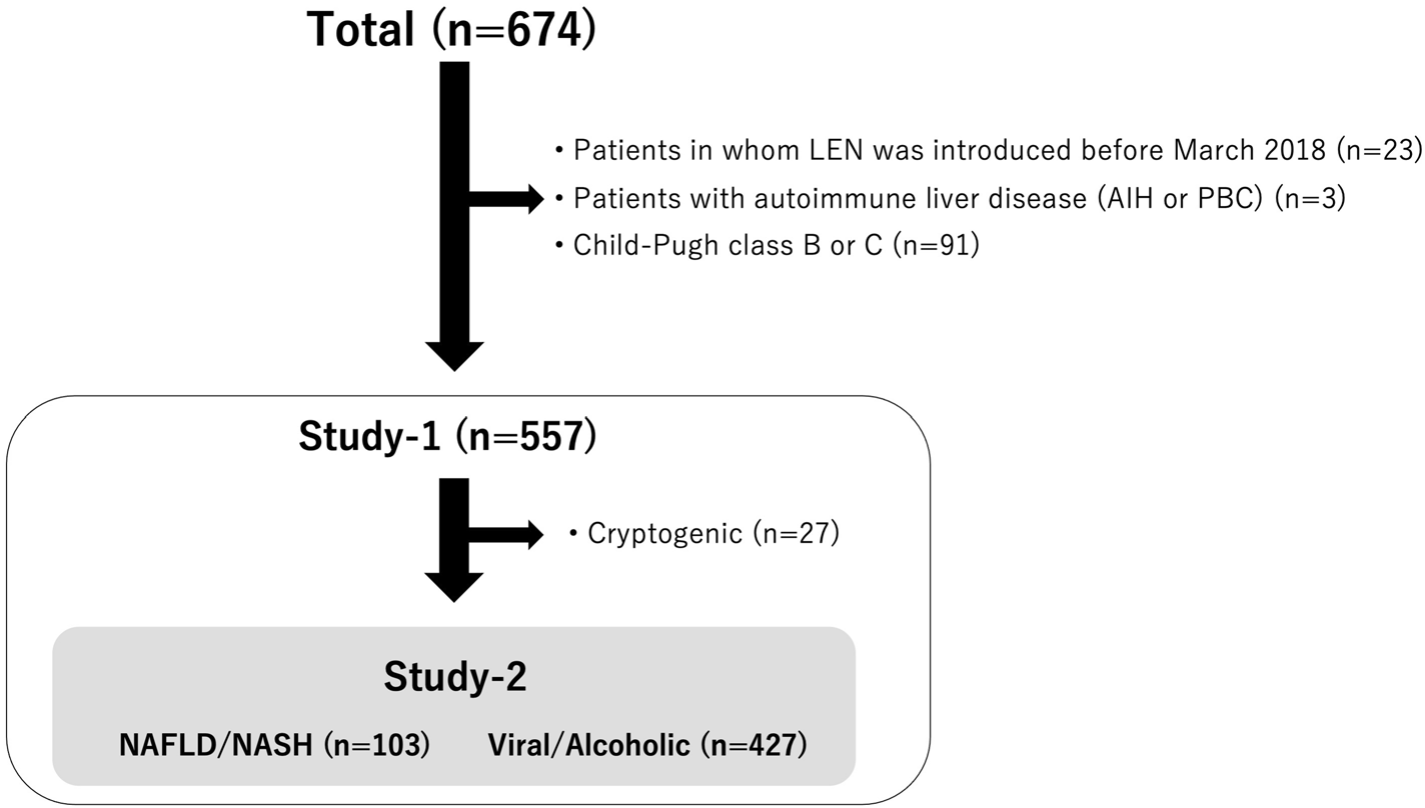
Flow of patient enrollment. *LEN* lenvatinib, *AIH* autoimmune hepatitis, *PBC* primary biliary cirrhosis.

The records of 674 patients with u-HCC and treated with lenvatinib at various institutions in Japan between March 2018 and February 2021 (Ehime Prefectural Central Hospital, Kindai University Hospital, Himeji Red Cross Hospital, Kagawa University Hospital, Okayama City Hospital, Osaka Medical School, Nippon Medical School, Ehime University Graduate Hospital, Teine Keijinkai Hospital, Saiseikai Niigata Hospital, Kagawa Prefectural Central Hospital, Asahi General Hospital, Toyama University Hospital, Otakanomori Hospital, Tokushima Prefectural Central Hospital, Matsuyama Red Cross Hospital, Hamamatsu University School of Medicine Hospital, Ogaki Municipal Hospital) were obtained. Those in whom lenvatinib was introduced before March 2018 as part of a clinical trial (n = 23), classified as Child–Pugh class B or C (n = 91), or with autoimmune liver disease [autoimmune hepatitis (AIH) or primary biliary cirrhosis (PBC)] (n = 3) were excluded, thus 557 cases were subjected to evaluations performed in a retrospective manner (Fig. 1).

Patients positive for hepatitis B virus surface antigen (HBsAg) were judged to have HCC due to the presence of hepatitis B virus (HBV), while those positive for anti-hepatitis C virus (HCV) were judged to have HCC due to HCV. Two patients positive for both HCV and HBV were included in the HCV group for this study because HBV DNA levels were below the detection level. For patients with a history of alcohol abuse of 60 g/day or more^6,7^, background liver disease was judged as alcoholic. NAFLD/NASH diagnosis was determined by a medical interview [history of obesity, hyperlipidemia, hypertension, etc., and/or no/low alcohol intake (< 30 g/day in males, < 20 g/day in females)] of fatty liver patients and/or based on pathological findings^8^. Burned-out NASH liver cirrhosis was diagnosed clinically based on the clinical course (e.g., no history of alcohol abuse, history of obesity and/or fatty liver, or past pathological diagnosis) by each institution. Patients without autoimmune liver disease (AIH or PBC) other than the above, or those in whom hepatic fibrosis was not observed pathologically were classified as cryptogenic liver disease. Positive for severe fibrosis was defined based on elevated FIB-4 index (≥ 3.25)^9^.

The therapeutic effects of lenvatinib in all 557 patients with Child–Pugh class A were examined as Study-1. Furthermore, therapeutic responses were compared between patients with NAFLD/NASH (n = 103), and those with chronic hepatic viral infection or alcohol abuse (Viral/Alcohol group) (n = 427), after exclusion of cryptogenic patients (n = 27), as Study-2 (Fig. 1).

### HCC diagnosis

HCC was diagnosed based on an increasing trend of alpha-fetoprotein (AFP), as well as typical findings obtained in dynamic CT^10^, MRI^11,12^, and contrast enhanced ultrasonography (CEUS) with perflubutane (Sonazoid®, Daiichi Sankyo Co., Ltd., Tokyo, Japan) examinations^13,14^, and/or pathological findings. To evaluate tumor progression, Barcelona Clinic Liver Cancer (BCLC) stage^15^ and tumor node metastasis (TNM) stage were used, and determined as previously reported in a study for TNM staging of HCC conducted by the Liver Cancer Study Group of Japan (LCSGJ) 6th edition (TNM-LCSGJ)^16^.

### Assessment methods for hepatic reserve function and therapeutic response

Child–Pugh classification^17^ and albumin-bilirubin (ALBI) grade were used for assessment of hepatic reserve function^18–20^. To perform more detailed evaluations of patients with the middle ALBI grade of 2, a revised grading system was used that consisted of four levels, with sub-grading for the middle grade of 2 (2a and 2b) based on an ALBI score of − 2.27 as the cut-off (modified ALBI, mALBI grade), which was previously reported to result in a predictive value for indocyanine green retention after 15 min (ICG-R15) of 30%^21,22^. Progression-free survival (PFS) was analyzed according to the modified Response Evaluation Criteria In Solid Tumors (mRECIST) criteria^23,24^, based on results of dynamic CT examinations performed at intervals of 8–12 weeks.

### Lenvatinib treatment and assessment of adverse events

After obtaining written informed consent from each patient, lenvatinib treatment was started. The drug was orally administered at 8 mg/day in patients weighing < 60 kg or 12 mg/day in those ≥ 60 kg, and discontinued when any unacceptable or serious adverse event (AE) occurred (any grade 3 or more severe AE, or any unacceptable grade 2 drug-related AE), or radiological tumor progression was observed, according to the guidelines for administration of lenvatinib. AEs were assessed according to the National Cancer Institute Common Terminology Criteria for Adverse Events, version 4.0^25^. When a drug-related AE was noted, dose reduction or temporary interruption was maintained until the symptom was resolved to grade 1 or 2, according to the guidelines provided by the manufacturer. AEs of grade 3 or more were defined as severe, and the worst grade for each AE during the present observation period was recorded.

### Ethical approval

Written informed consent for lenvatinib treatment was obtained from each patient. This was a retrospective analysis of records stored in a database and official approval was received based on the Guidelines for Clinical Research issued by the Ministry of Health and Welfare of Japan. All procedures complied with the declaration of Helsinki. The study protocol was granted approval by the Institutional Ethics Committee of Ehime Prefectural Central Hospital (IRB No. 30-66) (UMIN000043219).

### Statistical analysis

Continuous variables are expressed as median values (first-third quartile). Statistical analyses were performed using Welch’s t-test, Student’s t-test, Fischer’s exact test, or Mann–Whitney’s *U* test, as appropriate. Cox hazard analysis (stepwise regression method), the Kaplan–Meyer method, and a log-rank test were used to analyze prognosis factors.

A P value less than 0.05 was considered to indicate statistical significance. All statistical analyses were performed using Easy R (EZR) version 1.53 (Saitama Medical Center, Jichi Medical University, Saitama, Japan)^26^, a graphical user interface for R (The R Foundation for Statistical Computing, Vienna, Austria).

## Results

### Study 1

**Figure 2:**
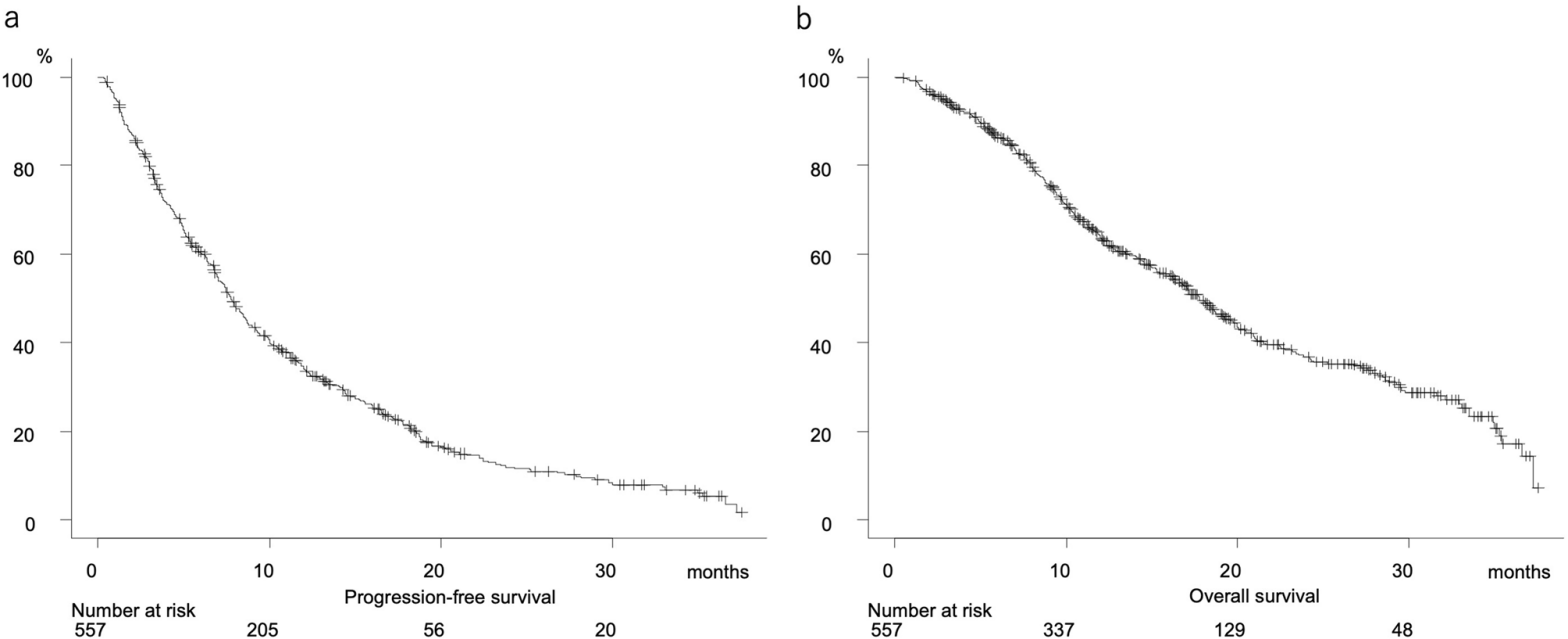
Progression-free and overall survival for all unresectable hepatocellular carcinoma patients with Child–Pugh class A (n = 557). (**a**) Progression-free survival (median 7.8 months, 95% CI 7.0–8.6). b. Overall survival (median 17.8 months, 95% CI 16.3–19.5).

**Figure 3:**
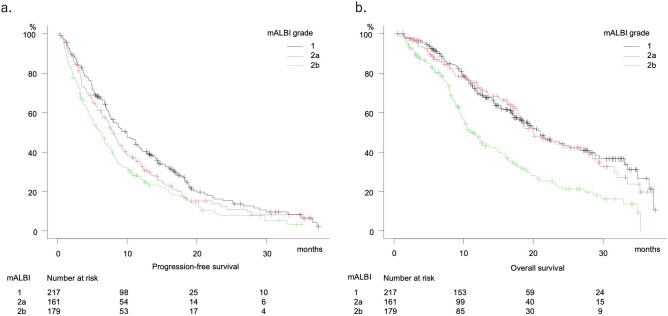
Progression-free and overall survival according to modified ALBI grade. (**a**) Progression-free survival divided by mALBI grade. mALBI 1 (median 9.8 months, 95% CI 7.8–11.6), mALBI 2a (median 8.0 months, 95% CI 6.6–9.3), mALBI 2b (6.3 months, 95% CI 4.8–7.4) (P = 0.002). (**b**) Overall survival divided by mALBI grade. mALBI 1 (median 21.0 months, 95% CI 17.2–26.8), mALBI 2a (median 20.0 months, 95% CI 17.8–27.9), mALBI 2b (median 11.2 months, 95% CI 9.7–14.5) (P < 0.001).

Clinical features of all 557 patients are shown in Table 1. PFS and OS were 7.8 months (95%CI 7.0–8.6 months) and 17.8 months (95%CI 16.3–19.5 months), respectively (Fig. 2a,b), and were well stratified according to mALBI grade (median PFS and OS: grade 1:2a:2b = 9.8:8.0:6.3 months, P = 0.002, and 21.0:20.0:11.2 months, P < 0.001, respectively) (Fig. 3a,b), while there were no significant differences for those according to treatment line (first, second, third or greater) using lenvatinib (median PFS and OS: 7.6:8.2:7.7 months, P = 0.080, and 16.7:18.3:23.2 months, P = 0.091, respectively) (Supplemental Fig. S1a,b)]. A comparison between initial and later line (second or greater) showed no significant difference regarding PFS (7.6 vs. 8.1 months, P = 0.752), while a significant difference was noted for OS (16.7 vs. 19.6 months, P = 0.029) (Supplemental Fig. S1c,d).

**Table 1:** Clinical features of all u-HCC patients.

	n = 557
Age, years*	73.0 (67.0 to 79.0)
Gender, male:female	430:127
Etiology, HCV:HBV:alcohol:NAFLD/NASH:cryptogenic	236:88:103:103:27
ECOG PS, 0:1:2:3	474:73:9:1
Body mass index (kg/m^2^)	22.98 (20.74 to 25.55)
ALBI score*	− 2.49 (− 2.18 to − 2.74)
(mALBI grade 1:2a:2b)	(217:161:179)
Child–Pugh score, 5:6	359:198
AFP, ≥ 400 ng/mL (%)	167 (30.0%)
TNM-LCSGJ, I:II:III:IVa:IVb	6:74:207:81:189
BCLC stage, 0:A:B:C:D	4:10:221:321:1
Lenvatinib treatment line, first:second:third:fourth:fifth	355:132:63:6:1
Deaths (%)	301 (54.0%)
Observation period, months	12.2 (6.9 to 19.2)

*HCV* hepatitis C virus, *HBV* hepatitis B virus, *NAFLD*: non-alcoholic fatty liver disease, *NASH* non-alcoholic steatohepatitis, *ECOG PS* Eastern Cooperative Oncology Group performance status, *ALBI score* albumin-bilirubin score, *mALBI grade* modified ALBI grade, *AFP* alpha-fetoprotein, *TNM LCSGJ 6th* tumor node metastasis stage by Liver Cancer Study Group of Japan 6th edition, *BCLC stage* Barcelona Clinic Liver Cancer stage.

*Median (interquartile range).

**Figure 4:**
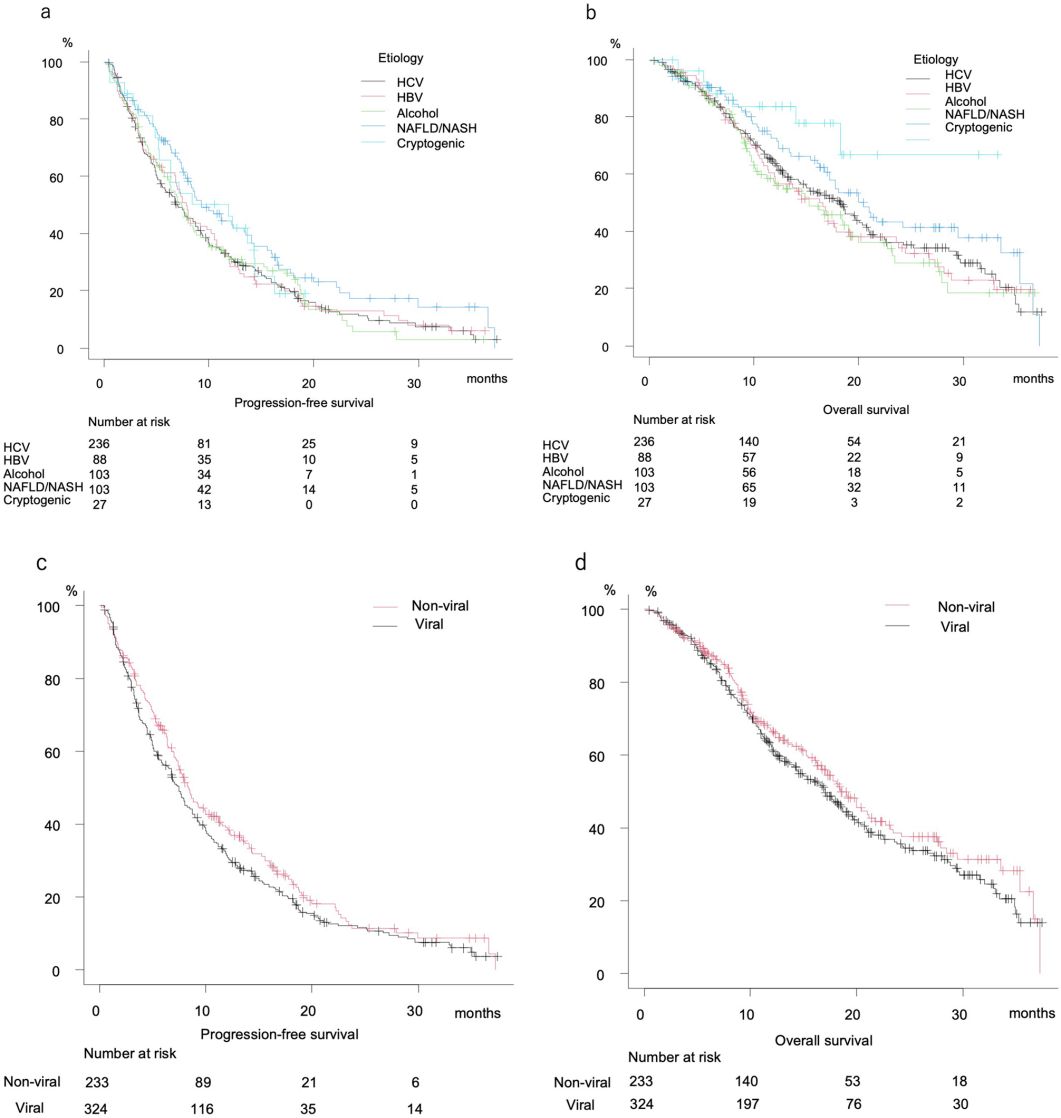

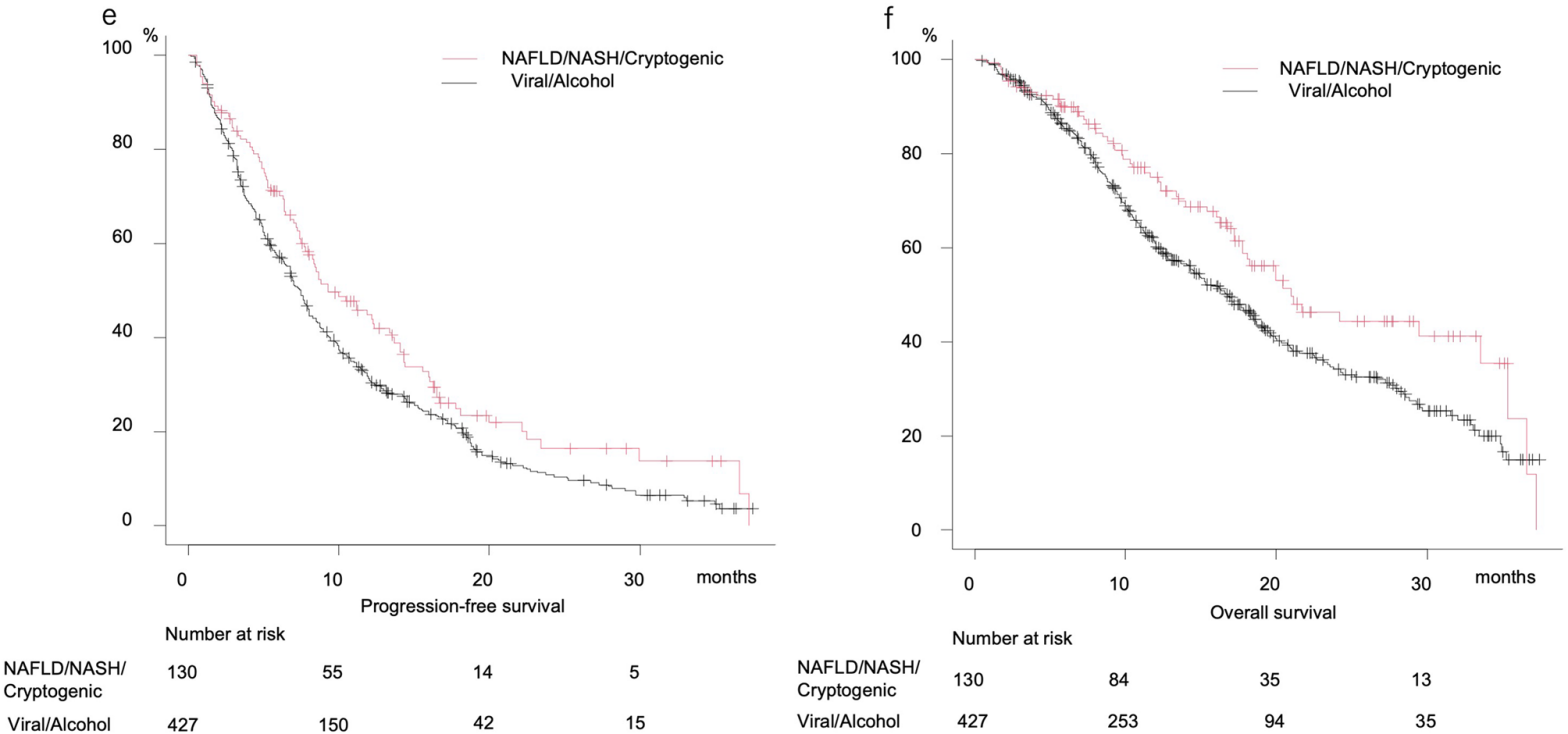
Progression-free and overall survival according to basal liver disease etiology. (**a**) Progression-free survival divided by basal liver disease etiology. HCV (median 7.0 months, 95% CI 5.5–8.7), HBV (median 7.9 months, 95% CI 6.8–10.5), alcohol (median 7.4 months, 95% CI 5.8–8.6), NAFLD/NASH (median 9.3 months, 95% CI 7.8–13.5), cryptogenic (median 11.9 months, 95% CI 5.2–14.4) (P = 0.154). (**b**) Overall survival divided by basal liver disease etiology. HCV (median 18.3 months, 95% CI 14.3–20.4), HBV (median 16.3 months, 95% CI 11.4–19.3), alcohol (median 15.3 months, 95% CI 10.5–19.2), NAFLD/NASH (median 20.5 months, 95% CI 16.8–29.5), cryptogenic (median not reached, 95% CI 18.3-not reached) (P = 0.052). (**c**) Progression-free survival of Viral and Non-viral (NAFLD/NASH, Cryptogenic, Alcohol) groups. Viral (median 7.5 months, 95% CI 6.4–8.5), Non-viral (median 8.3 months, 95% CI 7.4–10.0) (P = 0.092). (**d**) Overall survival of Viral and Non-viral (NAFLD/NASH, Cryptogenic, Alcohol) groups. Viral (median 17.2 months, 95% CI 14.4–19.3), Non-viral (median 18.5 months, 95% CI 16.3–21.7) (P = 0.226). (**e**) Progression-free survival of Viral/Alcohol and NAFLD/NASH/Cryptogenic groups. Viral/Alcohol (median 7.5 months, 95% CI 6.8–8.0), NAFLD/NASH/Cryptogenic (median 9.3, 95% CI 7.8–13.4) (P = 0.012). (**f**) Overall survival of Viral/Alcohol and NAFLD/NASH/Cryptogenic groups. Viral/Alcohol (median 16.9 months, 95% CI 14.5–18.6), NAFLD/NASH/Cryptogenic (median 21.0 months, 95% CI 17.8–33.5) (P = 0.009).

Median PFS after dividing patients into HCV, HBV, alcohol, NAFLD/NASH, and cryptogenic groups was 7.0, 7.9, 7.4, 9.3, and 11.9 months, respectively, (P = 0.154) (Fig. 4a), while median OS was 18.3, 16.3, 15.3, 20.5 months, and not reached, respectively (P = 0.052) (Fig. 4b). There were no significant differences in regard to PFS (7.5 vs. 8.3 months, P = 0.092) or OS (17.2 vs. 18.5 months, P = 0.226) between the viral HCC and nonviral (NAFLD/NASH, cryptogenic, alcohol) groups (Fig. 4c,d). Since median PFS and OS for the alcohol group were similar to those for the viral group, patients associated with alcohol abuse were included in the viral group, and comparisons between the Viral/Alcohol group and others (NAFLD/NASH, cryptogenic) were performed. Those results showed that both PFS and OS for the Viral/Alcohol group were significantly worse (median PFS: 7.5 vs. 9.3 months, P = 0.012; median OS: 16.9 vs. 21.0 months, P = 0.009) (Fig. 4e,f).

### Study-2

In comparisons between the NAFLD/NASH and Viral/Alcohol groups, platelet count and FIB-4 index were better in the former (15.3 × 10^4^/µL vs. 13.1 × 10^4^/µL and 3.81 vs. 4.39, P = 0.005 and P = 0.038, respectively). On the other hand, a larger percentage of patients in the NAFLD/NASH group started lenvatinib at a reduced dose (36.9% vs. 26.0%, P = 0.037), while there was no significant difference observed in regard to hepatic function (Child–Pugh score, ALBI score, mALBI grade), tumor burden (TNM-LCSGJ, BCLC stage), or malignancy grade of HCC (elevated AFP: ≥ 400 ng/mL) between them (Table 2).

**Table 2:** Comparison of clinical features between NAFLD/NASH and Viral/Alcohol groups after exclusion of cryptogenic HCC.

	NAFLD/NASH (n = 103)	Viral/alcohol (n = 427)	P value
Age, years *	75 (69 to 80)	73 (66 to 79)	0.078
Gender, male:female	77:26	334:93	0.434
Etiology, HCV:HBV:alcohol:NAFLD/NASH	0:0:0:103	236:88:103:0	< 0.001
ECOG PS, 0:1:2	89:11:3	364:58:5	0.270
Body mass index, kg/m^2^	24.93 (22.79 to 27.41)	22.65 (20.32 to 25.04)	< 0.001
Platelets, 10^4^/µL*	15.3 (11.2 to 20.0)	13.1 (9.5 to 17.6)	0.005
AST, U/L*	40 (28 to 57)	41 (29 to 62)	0.273
ALT, U/L*	28 (21 to 41)	30 (19 to 47)	0.403
T-bilirubin, mg/dL*	0.68 (0.50 to 0.90)	0.70 (0.54 to 1.00)	0.094
Albumin, g/dL*	3.70 (3.50 to 4.00)	3.80 (3.45 to 4.10)	0.998
Prothrombin time, %*	89.0 (84.0 to 101.0)	88.0 (80.0 to 97.0)	0.142
eGFR, nL/min/1.73 m^2^*	66.0 (50.2 to 77.3)	66.4 (56.0 to 79.4)	0.367
ALBI score*	− 2.48 (− 2.21 to − 2.48)	− 2.48 (− 2.17 to − 2.76)	0.603
mALBI grade 1:2a:2b:3	41:31:31:0	164:123:140:0	0.869
Child–Pugh score, 5:6	72:31	269:158	0.208
AFP, ≥ 400 ng/mL	25 (24.3%)	136 (31.9%)	0.152
MVI (portal vein), none:Vp1:Vp2:Vp3:Vp4	89:1:4:7:2	350:12:29:24:12	0.663
MVI (hepatic vein), none:Vv1:Vv2:Vv3	90:8:4:1	391:22:9:5	0.417
Positive for EHM	40 (38.8%)	140 (32.8%)	0.249
TNM-LCSGJ, I:II:III:IVa:IVb	2:12:37:12:40	4:60:157:66:140	0.537
BCLC stage, 0:A:B:C	1:2:39:61	3:8:171:245	0.933
Initial dose of lenvatinib, 4:8:12 mg*	5:55:43	36:240:151	0.174
Reduced starting dose	38 (36.9%)	111 (26.0%)	0.037
Lenvatinib treatment line: first:second:third:fourth:fifth	73:18:11:0:1	262:108:51:6:0	0.093
FIB-4 index	3.81 (2.31 to 5.55)	4.39 (2.80 to 6.34)	0.038
Pathological diagnosis of NAFLD/NASH during clinical course (%)	27 (26.2%)	NA	NA
Deaths (%)	51 (49.5%)	244 (57.1%)	0.185
Observation period, months	13.5 (7.5 to 21.3)	11.9 (6.8 to 18.9)	0.124

*HCV* hepatitis C virus, *HBV* hepatitis B virus, *NAFLD* non-alcoholic fatty liver disease, *NASH* non-alcoholic steatohepatitis, *ECOG PS* Eastern Cooperative Oncology Group performance status, *AST* aspartate transaminase, *ALT* alanine aminotransferase, *ALBI score* albumin-bilirubin score, *mALBI grade* modified ALBI grade, *AFP* alpha-fetoprotein, *MVI* macrovascular invasion, *EHM* extra-hepatic metastasis, *TNM LCSGJ 6th* tumor node metastasis stage by Liver Cancer Study Group of Japan 6th edition, *BCLC stage* Barcelona Clinic Liver Cancer stage, *NA* not applicable.

*Median (interquartile range).

**Figure 5:**
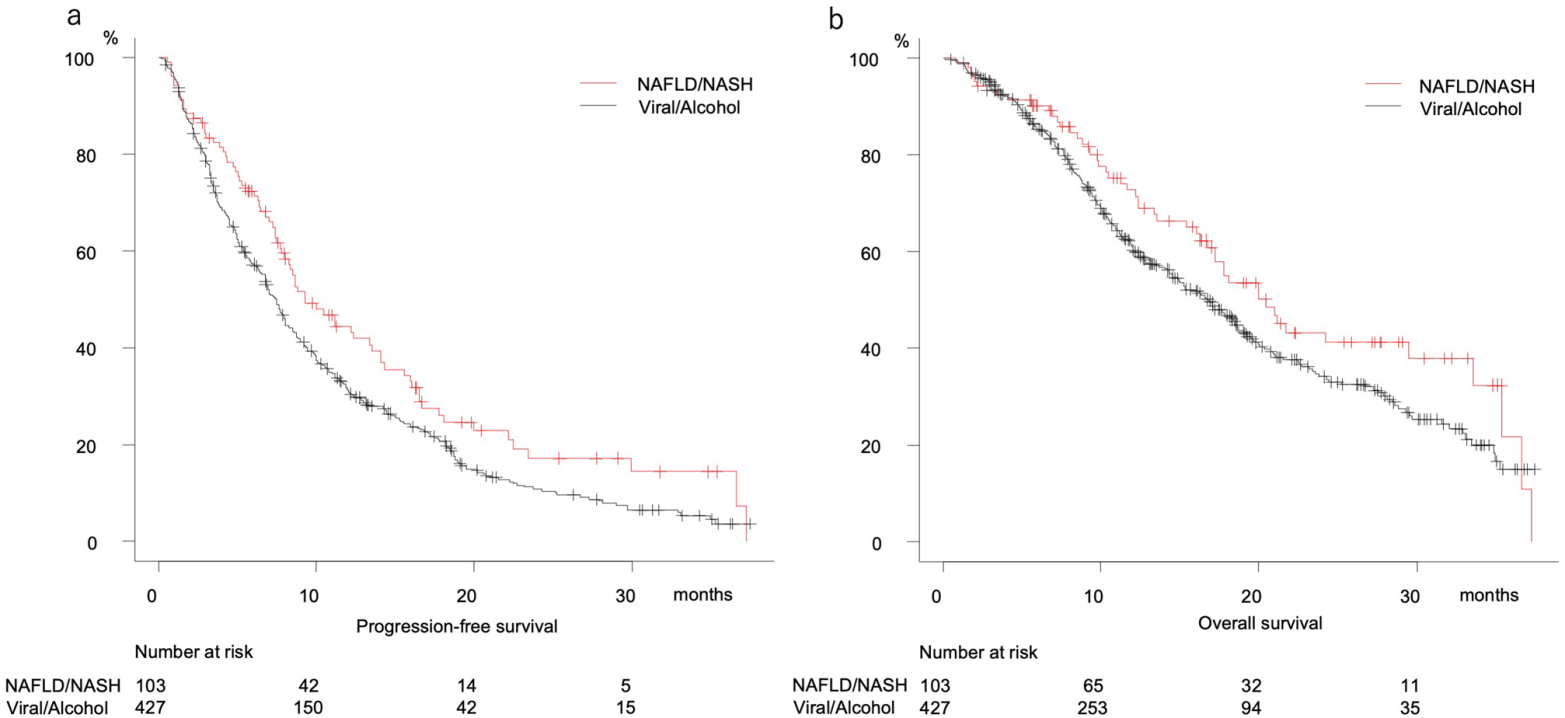
Progression-free and overall survival of NAFLD/NASH and Viral/Alcohol groups. (**a**) Progression-free survival. Viral/Alcohol (median 7.5 months, 95% CI 6.8–8.0), NAFLD/NASH (median 9.3 months, 95% CI 7.8–13.5) (P = 0.012). (**b**) Overall survival. Viral/Alcohol (median 16.9 months, 95% CI 14.5–18.6), NAFLD/NASH (median 20.5 months, 95% CI 16.8–29.5). (P = 0.057).

Following exclusion of cryptogenic patients, PFS was better in the NAFLD/NASH than the Viral/Alcohol group (median 9.3 vs. 7.5 months, P = 0.012) (Fig. 5a), while there was no significant difference in regard to OS (median 20.5 vs. 16.9 months, P = 0.057) (Fig. 5b). Cox-hazard analysis for prognostic factors of PFS showed elevated ALT (≥ 30 U/L) (HR 1.247, P = 0.029), mALBI grade 2b (HR 1.236, P = 0.047), elevated AFP (≥ 400 ng/mL) (HR 1.294, P = 0.014), and NASH/NAFLD (HR 0.763, P = 0.036) to be significant prognostic factors (Table 3a). Although NAFLD/NASH was not a significant prognostic factor in that analysis for OS (HR 0.758, P = 0.092), the factors AFP (≥ 400 ng/mL) (HR 1.402, P = 0.009), BCLC C stage (HR 1.297, P = 0.035), later line introduction of lenvatinib (HR 0.737, P = 0.014), and mALBI grade 2b (HR 1.875, P < 0.001) were significant (Table 3b). Additionally, FIB-4 index was lower in the NAFLD/NASH group, though elevated FIB-4 index (≥ 3.25) was not a significant prognostic factor in regard to either PFS or OS (Table 3a,b).

**Table 3:** Prognostic factors for progression-free survival and overall survival in Study-2.

a. Cox hazard analysis for PFS	HR	95%CI	P value
Age ≥ 75 years	1.059	0.863–1.300	0.583
Female gender	1.013	0.802–1.280	0.912
ECOG PS 2	0.946	0.377–2.374	0.906
NAFLD/NASH	0.730	0.562–0.978	0.019
ALT ≥ 30 U/L	1.240	1.022–1.522	0.030
Platelet count ≥ 10 (10^4^/µL)	0.935	0.732–1.194	0.588
Elevated FIB-4 index (≥ 3.25)	1.122	0.892–1.412	0.326
mALBI grade 2b	1.172	0.945–1.453	0.148
AFP ≥ 400 ng/mL	1.272	1.031–1.569	0.025
TNM-LCSGJ stage IV	1.194	0.818–1.742	0.358
BCLC stage C	1.043	0.713–1.526	0.827
Reduced starting dose	1.215	0.980–1.505	0.076
Treatment as later line	0.880	0.718–1.079	0.219
Results of stepwise regression method			
NAFLD/NASH	0.763	0.594–0.982	0.036
ALT ≥ 30 U/L	1.247	1.023–1.520	0.029
mALBI grade 2b	1.236	1.003–1.523	0.047
AFP ≥ 400 ng/mL	1.294	1.055–1.588	0.014

b. Cox hazard analysis for OS	HR	95%CI	P value
Age ≥ 75 years	1.167	0.904–1.505	0.236
Female gender	1.141	0.862–1.510	0.358
ECOG PS 2	1.106	0.394–3.105	0.848
NAFLD/NASH	0.758	0.550–1.046	0.092
ALT ≥ 30 U/L	1.188	0.934–1.512	0.160
Platelet count ≥ 10 (10^4^/µL)	1.038	0.772–1.396	0.805
Elevated FIB-4 index (≥ 3.25)	1.102	0.808–1.468	0.506
mALBI grade 2b	1.764	1.376–2.262	< 0.001
AFP ≥ 400 ng/mL	1.367	1.059–1.765	0.016
TNM-LCSGJ stage IV	1.183	0.762–1.837	0.454
BCLC stage C	1.174	0.756–1.824	0.475
Reduced starting dose	1.055	0.807–1.380	0.694
Treatment as later line	0.737	0.574–0.946	0.017
Results of stepwise regression method			
mALBI grade 2b	1.875	1.481–2.375	< 0.001
AFP ≥ 400 ng/mL	1.402	1.089–1.805	0.009
BCLC stage C	1.297	1.019–1.652	0.035
Treatment as later line	0.737	0.578–0.939	0.014

*ALT* alanine aminotransferase, *NAFLD* non-alcoholic fatty liver disease, *NASH* non-alcoholic steatohepatitis, *ECOG PS* Eastern Cooperative Oncology Group performance status, ALBI score: albumin-bilirubin score, *mALBI grade* modified ALBI grade, *AFP* alpha-fetoprotein, *TNM LCSGJ 6th* tumor node metastasis stage by Liver Cancer Study Group of Japan 6th edition, *BCLC stage* Barcelona Clinic Liver Cancer stage.

Finally, examination of AEs (over 20%) showed that hypothyroid and urine protein conditions were more common in the NAFLD/NASH as compared to the Viral/Alcohol group (P = 0.013 and P = 0.032, respectively) (Supplemental Table S1).

## Discussion

The present analyses of u-HCC patients who received lenvatinib showed that PFS and OS in those in the NAFLD/NASH group were favorable as compared with those in the Viral/Alcohol group (median PFS: 9.3 vs. 7.5 months, P = 0.012) (median OS: 20.5 vs. 16.9 months, P = 0.057). Also, when cryptogenic HCC was included in the NAFLD/NASH group, both PFS and OS were better in those patients (median PFS: 9.3 vs. 7.5 months, P = 0.012) (median OS: 21.0 vs. 16.9 months, P < 0.001). An interesting meta-analysis article recently reported by Pfister showed that patients with a viral etiology demonstrated therapeutic benefits with ICI treatment [HR 0.64], whereas those with nonviral etiology HCC did not [HR 0.92] (P = 0.03)^5^. That report also presented results of two different validation studies of ICI treatment for HCC, in which NAFLD-HCC cases showed significantly worse OS than cases of HCC with another etiology (HR 2.6, 95% CI 1.2–5.6, P = 0.017; median 8.8 vs. 17.7 months, P = 0.034). In patients undergoing ICI treatment, background liver disease etiology might be a biomarker of efficacy. As for a reason for that phenomenon, it has been reported that CD8 + T cells in HCC patients with NASH are increased and activated by IL-15-induced Fas-ligand dependent apoptosis through tumor necrotic factor (TNF) and acetate in the tumor, unlike MHC class-I dependent CD8 + T cell activation^27^, thus immune response to tumor antigens is impaired^28,29^. In contrast, the effectiveness of MTA is not related with mode of CD8 + T cell activation, but rather inhibition of multi-tyrosine kinase activity, thus MTA should be effective irrespective of HCC etiology including in NAFLD/NASH-related HCC cases.

Patients in the IMbrave150 study who underwent treatment with Atezo + Bev, a newly developed ICI and anti-VEGF-antibody combination, showed an overwhelmingly superior therapeutic efficacy as compared with those who received sorafenib (median OS: 19.2 vs. 13.4 months, HR 0.66, 95%CI 0.52–0.85) (ORR/CR by mRECIST: 35%/12% vs. 14%/3%)^30^. Although pooled analysis of the SHARP and Asia–Pacific trials found that positive for HCV was a predictive factor for therapeutic response to sorafenib [HR 0.47, 95% CI 0.32–0.69, P = 0.035]^31^, the IMbrave150 study showed superiority for the therapeutic effect (both OS and PFS) of Atezo + Bev as compared with sorafenib in HCV-HCC cases (HR 0.43, 95% CI 0.25–0.73 and HR 0.68, 95% CI 0.42–1.10, respectively)^30^. On the other hand, that study did not demonstrate superior findings for Atezo + Bev in regard to OS in HCC with nonviral etiology (HR 1.05, 95% CI 0.68–1.63 and HR 0.80, 95% CI 0.55–1.17) as compared to viral HCC cases. However, these results do not indicate that Atezo + Bev is not effective for non-viral HCC, as the OS in patients who received that treatment was 17.0 months, similar to that in patients with HBV HCC (19.0 months). Rather, the worse OS HR can be attributed to better efficacy of sorafenib even in non-viral HCC (18.1 months) as compared with HBV-HCC (12.4 months) and HCV-HCC (12.6 months) cases, though the reasons are unknown. The present results suggested a similar phenomenon. Although liver fibrosis in the background of HCC patients with NAFLD/NASH may be milder as compared to that in those with Viral/Alcohol, elevated FIB-4 index was not significant prognostic factor both in PFS and OS. An explanation for these findings is not clear.

Despite the retrospective nature of this analysis, it is possible to speculate that cryptogenic HCC is a subgroup of NAFLD/NASH HCC. In the present Study-1, patients with u-HCC due to alcohol abuse had PFS and OS similar to those with viral HCC, thus viral and alcoholic HCC were treated as a single group in comparisons of PFS and OS with those of NAFLD/NASH/cryptogenic HCC patients. In Study-2, after excluding cryptogenic HCC, the NAFLD/NASH and other etiology (Viral/Alcohol) groups were compared to confirm response to lenvatinib in clinically diagnosed NAFLD/NASH patients. The NAFLD/NASH group showed better PFS (P = 0.012). Although there was no significant difference in OS (P = 0.057), OS in the NAFLD/NASH-HCC patients treated with lenvatinib was very favorable (20.5 months) and tended to be better than that in the Viral/Alcohol HCC cases (16.9 months). It was recently proposed by Hessheier et al. that metabolic factors may be risk factors for development of liver diseases and cirrhosis^32^, while Eguchi et al. found “lean-NASH” (non-obese NASH, body mass index: BMI < 25 kg/m^2^) existing in 20% to > 35% in patients in Japan^33^. Of the present cryptogenic HCC patients (n = 26), diabetes was observed in 44.4% (n = 12), hypertension in 51.9% (n = 14), and overweight (BMI ≥ 25 kg/m^2^) in 25.9% (n = 7), while 70.4% (n = 19) had at least one of those co-factors (Supplemental Table S2). Thus, cryptogenic HCC might be categorized as NAFLD/NASH HCC. When cryptogenic HCC cases are included with NAFLD/NASH, in other words, without hepatitis viral infection or alcohol abuse history, such patients may receive benefit from lenvatinib treatment (Fig. 4e,f).

Since 2004, the number of adults with NASH awaiting liver transplantation in the United States has nearly tripled and NASH has become the second leading etiology of liver disease among such cases^34^. In meta-analysis results, the NAFLD incidence rate was reported to be 25.24% (all regions, 95% CI 22.1–28.65) and pooled overall NASH prevalence among biopsied NAFLD patients was estimated to be 59.10% (95%CI 47.55‐69.73), while the annual rate of liver carcinogenesis from NAFLD was estimated to be approximately 0.04% (95% CI 0.29–0.66)^35^. Similarly in Japan, a rapidly increasing rate of HCC patients without hepatitis viruses has been reported^36^, with most cases of non-B, non-C HCC shown to be related to lifestyle/metabolic factors, such as obesity or diabetes, including cryptogenic HCC^37^. Recently, liver-related diseases, such as cirrhosis and HCC, have been reported to be the third leading cause of death in patients in Japan with type 2 diabetes mellitus, which is associated with NAFLD^38^. Furthermore, a recent review article of cases of HCC related to NAFLD mentioned that the impact of metabolic syndrome and its relevance in those patients is not clear^39^. Nevertheless, establishment of an effective treatment strategy for u-HCC related with NAFLD/NASH is considered to be a critical clinical issue. It is anticipated that the number and percentage of NAFLD-HCC cases will continue to increase, though liver cirrhosis is not present in all of those. However, HCC is often detected in an advanced stage because no surveillance program for NAFLD-HCC patients has been established. As a result, it is important to confirm which systemic treatment (e.g. MTAs or ICI combination) is a more effective therapeutic option for patients with NAFLD HCC as well as those with viral hepatitis-related HCC. Moreover, some favorable results regarding OS in u-HCC patients receiving lenvatinib as post-progression treatment following ICI have been reported. Aoki noted that the median OS of lenvatinib was 15.8 months (95% CI 8.49–23.17) after ICI failure^40^, while Yoo reported that patients who received lenvatinib as post-progression treatment after Atez/Bev failure showed good OS (median 16.6 months)^41^. In the present analysis, lenvatinib showed a good therapeutic effect with both first and later line administration. Thus, not only for NAFLD/NASH u-HCC cases but also those with ICI treatment failure, lenvatinib can be selected for administration as an effective subsequent therapeutic option at any time, especially in patients with good hepatic function.

The present study has some limitations, including its design as a retrospective multicenter study. Furthermore, the pathological diagnosis of disease etiology for the present patients without viral hepatitis was not adequately assessed. A future study in which prospective comparisons between lenvatinib and ICI treatment in NASH/NAFLD HCC patients is needed.

In conclusion, lenvatinib was found to be effective for improving the prognosis of u-HCC patients irrespective of HCC etiology or line of treatment.

## Supplementary Information

Supplementary Information 1.

Supplementary Figure S1a,b.

Supplementary Figure S1c,d.

## Data Availability

The datasets generated and/or analyzed for the current study are not publicly available because of privacy reasons.
