# Supplementary material for: Efficacy of lenvatinib for unresectable hepatocellular carcinoma based on background liver disease etiology: multi-center retrospective study
Source: Sci Rep. 2021 Aug 17;11:16663. doi: 10.1038/s41598-021-96089-x (PMC8370989; doi:10.1038/s41598-021-96089-x)
Supplement: Supplementary file 1 — Supplementary Information 1. [file 41598_2021_96089_MOESM1_ESM.docx]

Supplemental Table 1. Comparison of adverse events (>20%) between NAFLD/NASH and Viral/Alcohol groups

|  | Total (n=527) | NAFLD/NASH  (n=103) | Viral/Alcohol  (n=427) | P value |
| --- | --- | --- | --- | --- |
| General fatigue (grade 1&2/3&4) | 68:66/21:2  (25.4%/4.4%) | 14:13/7:1  (26.2%/7.8%) | 54:53/14:1  (25.1%/3.5%) | 0.291 |
| Hypothyroid (grade 1&2/3) | 28:100/4  (24.3%/0.8%) | 12:21/1  (32.0%/1.0%) | 16:79/3  (22.2%/0.7%) | 0.013 |
| Hand-foot skin reaction (grade 1&2/3&4) | 61:52/14  (21.4%/2.7%) | 13:10/4  (22.3%/3.9%) | 48:42/10  (21.1%/2.3%) | 0.747 |
| Hypertension (grade 1&2/3) | 35:69/16  (19.7%/3.0%) | 7:14/3  (20.4%/2.9%) | 28:55/13  (19.4%/3.0%) | 0.989 |
| Appetite loss (grade 1&2/3) | 68:39/8  (20.3%/1.5%) | 11:21/7  (31.1%/6.8%) | 57:18/1  (17.6%/0.2%) | 0.208 |
| Urine protein (grade 1&2/3) | 45:39/29  (15.9%/5.5%) | 13:9/10  (21.4%/9.7%) | 32:30/19  (14.5%/4.4%) | 0.032 |

Supplemental Table 2. Clinical features of cryptogenic HCC patients

|  | n=27 | |
| --- | --- | --- |
| Age, years * | 76.0 (69.0-79.0) | |
| Gender, male:female | 19:8 | |
| ECOG PS, 0:1:2:3 | 21:4:1:1 | |
| Body mass index (kg/m^2^)  (overweight 25 kg/m^2^, %) | 22.44 (21.26 to 24.73)  (7, 25.9%) | |
| Platelets, 10^4^/µL * | 14.4 (11.3-18.7) | |
| AST, U/L * | 40 (27-47) | |
| ALT, U/L * | 27 (18-39) | |
| T-bilirubin, mg/dL * | 0.70 (0.55-0.90) | |
| Albumin, g/dL * | 3.80 (3.50-4.05) | |
| FIB-4 index | 3.72 (2.11-5.51) | |
| Positive for diabetes (%) | 12 (44.4%) | |
| Positive for hypertension (%) | 14 (51.8%) | |
| ALBI score *  (mALBI grade 1:2a:2b) | -2.56 (-2.22 to -2.76)  (12:7:8) | |
| Child-Pugh score, 5:6 | 18:9 | |
| AFP, ≥400 ng/mL (%) | 6 (28.6%) | |
| TNM-LCSGJ, II:III:IVa:IVb | 2:13:3:9 | |
| BCLC stage, B:C:D | 11:15:1 | |
| Lenvatinib treatment line: first:second:third | 20:6:1 | |
| Deaths (%) | 6 (28.6%) | |
| Observation period, months | 14.0 (7.3 to 17.9) |  |

*Median (interquartile range). HCV: hepatitis C virus, HBV: hepatitis B virus, NAFLD: non-alcoholic fatty liver disease, NASH: non-alcoholic steatohepatitis, ECOG PS: Eastern Cooperative Oncology Group performance status, AST: aspartate transaminase, ALT: alanine aminotransferase, ALBI score: albumin-bilirubin score, mALBI grade: modified ALBI grade, AFP: alpha-fetoprotein, TNM LCSGJ 6^th^: tumor node metastasis stage by Liver Cancer Study Group of Japan 6^th^ edition, BCLC stage: Barcelona Clinic Liver Cancer stage

Supplemental Figure S1. Progression-free and overall survival according to lenvatinib treatment line.

1. Progression-free survival divided by line of administration. First line (median 7.6 months, 95%CI: 6.8-8.7), second line (median 8.2 months, 95%CI: 6.9-11.9), third line (median 7.7 months, 95%CI: 5.0-10.0) (P=0.800).
2. Overall survival divided by line of administration. First line (median 16.7 months, 95%CI: 14.1-18.6), second line (median 18.3 months, 95%CI: 16.0-24.3), third line (median 23.2 months, 95%CI: 16.3-28.8) (P=0.091).
3. Progression-free survival divided by first and later line. First line (median 7.6 months, 95%CI: 6.8-8.7), later line (median 8.1 months, 95%CI: 7.0-9.5) (P=0.752).
4. Overall survival divided by first and later line. First line (median 16.7 months, 95%CI: 14.1-18.6), later line (median 19.6 months, 95%CI: 17.1-24.4) (P=0.029).
