# Supplementary figures and images for: Efficacy of lenvatinib for unresectable hepatocellular carcinoma based on background liver disease etiology: multi-center retrospective study
Source: Sci Rep. 2021 Aug 17;11:16663. doi: 10.1038/s41598-021-96089-x (PMC8370989; doi:10.1038/s41598-021-96089-x)

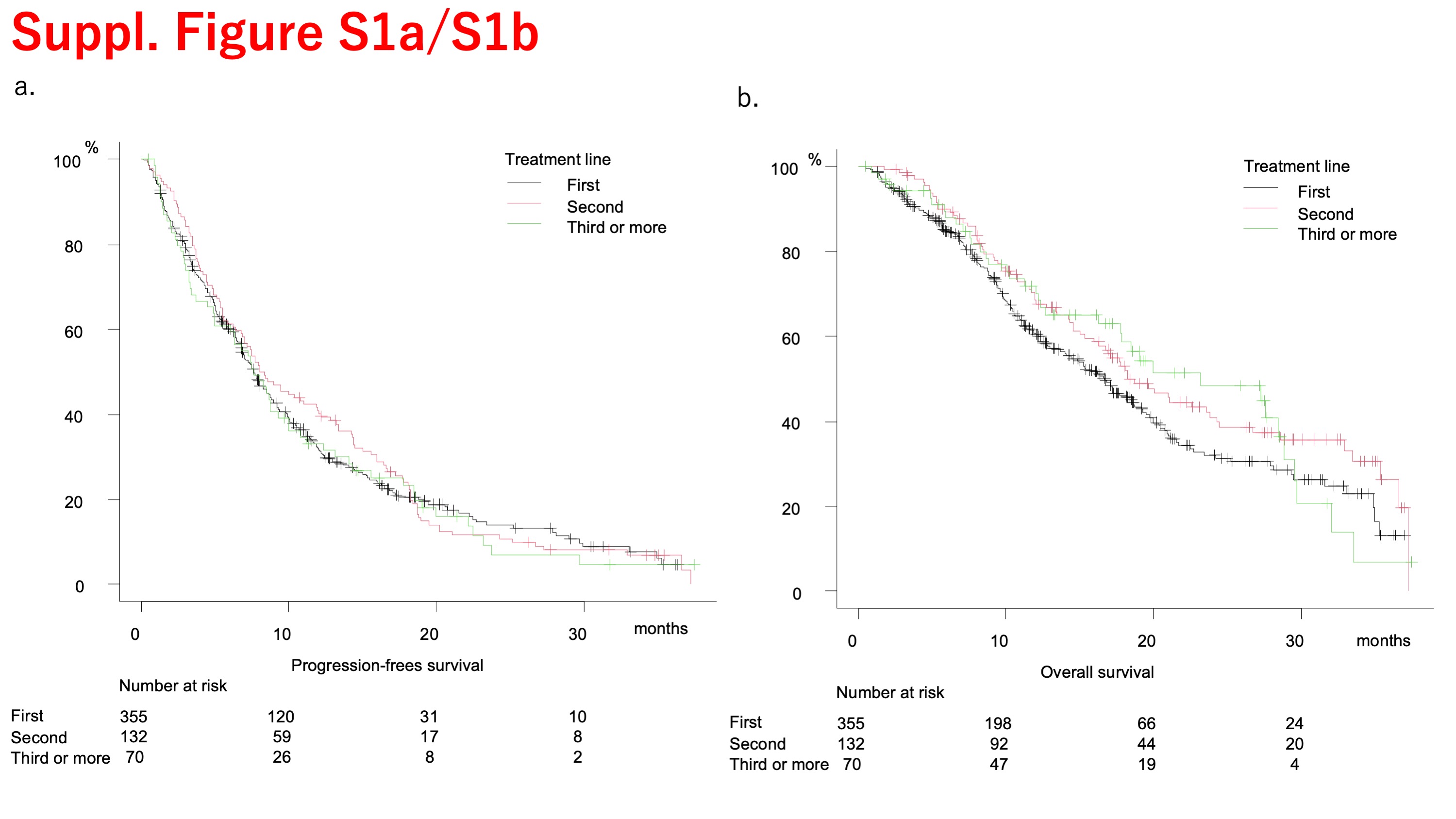

Supplement: Supplementary file 2 — Supplementary Figure S1a,b. [file 41598_2021_96089_MOESM2_ESM.jpeg]

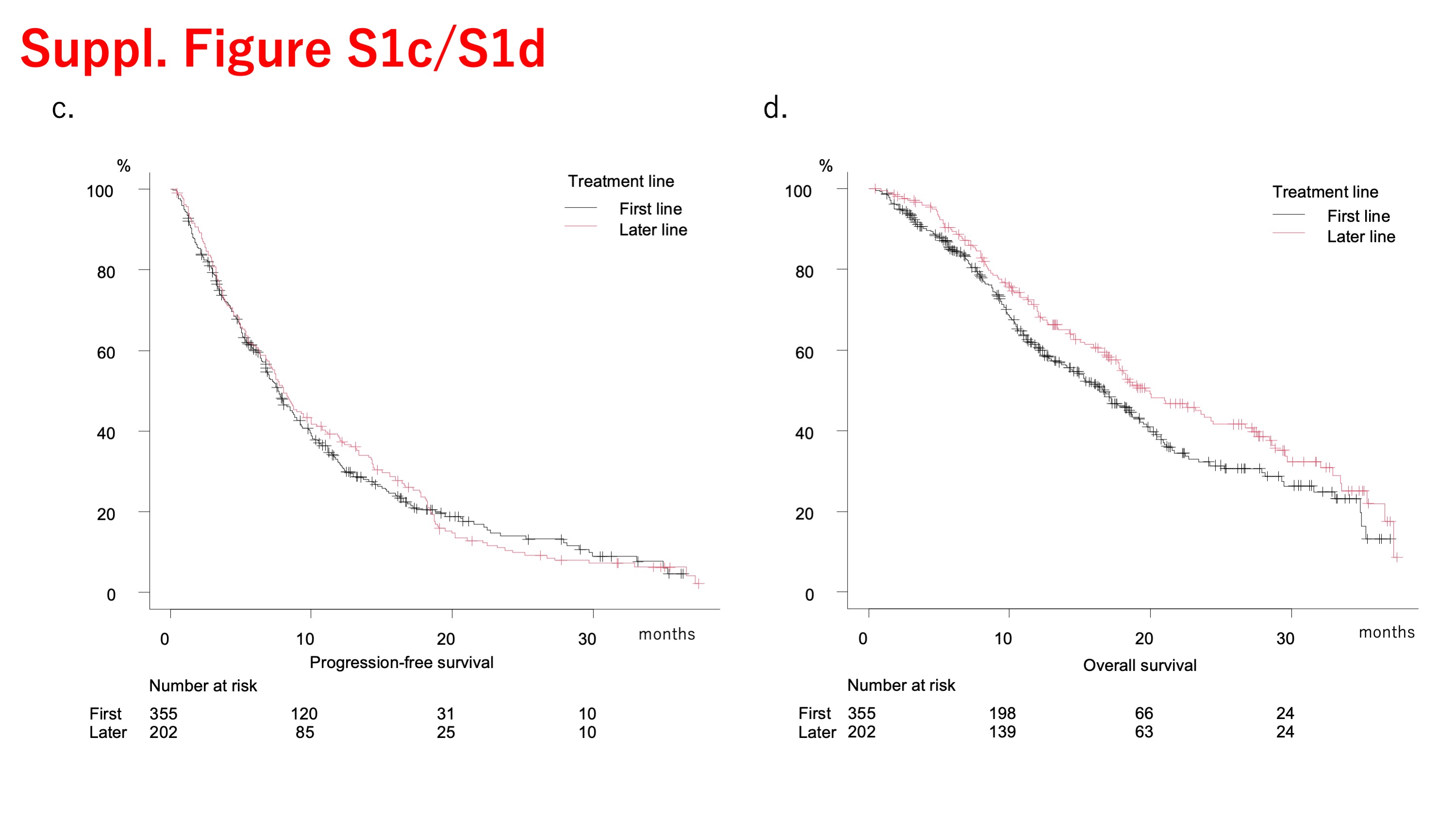

Supplement: Supplementary file 3 — Supplementary Figure S1c,d. [file 41598_2021_96089_MOESM3_ESM.jpeg]
